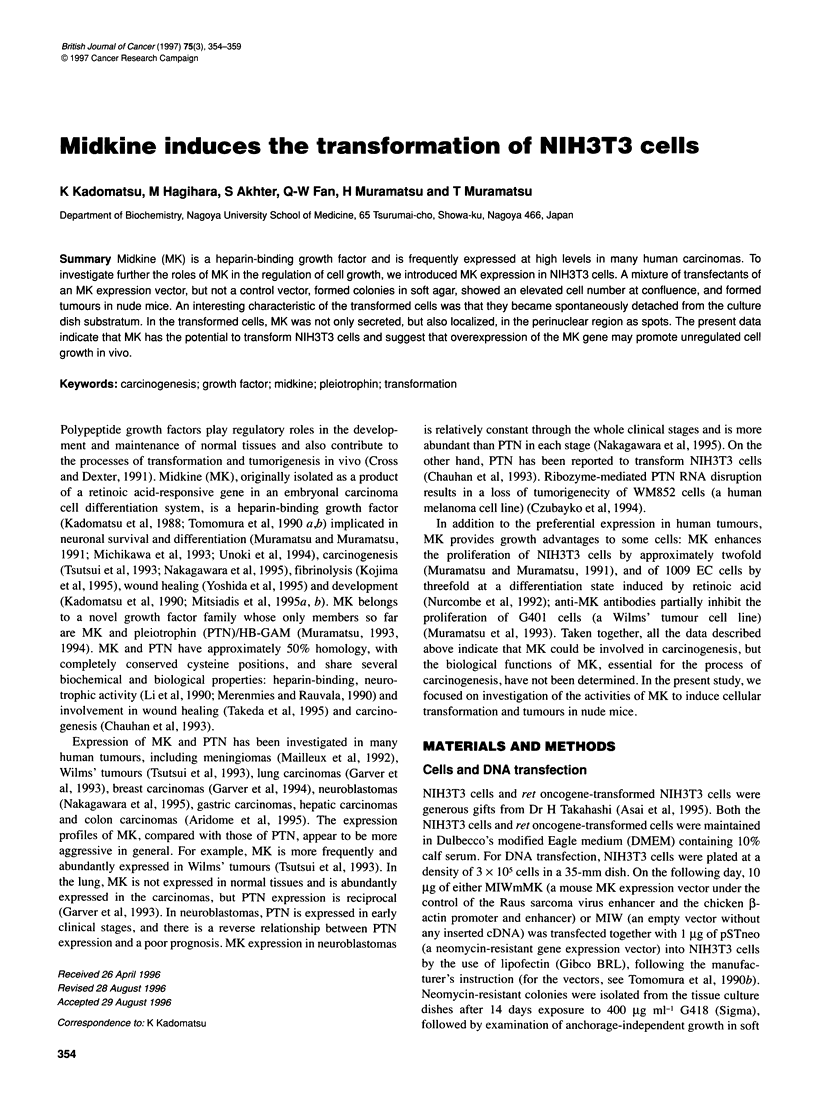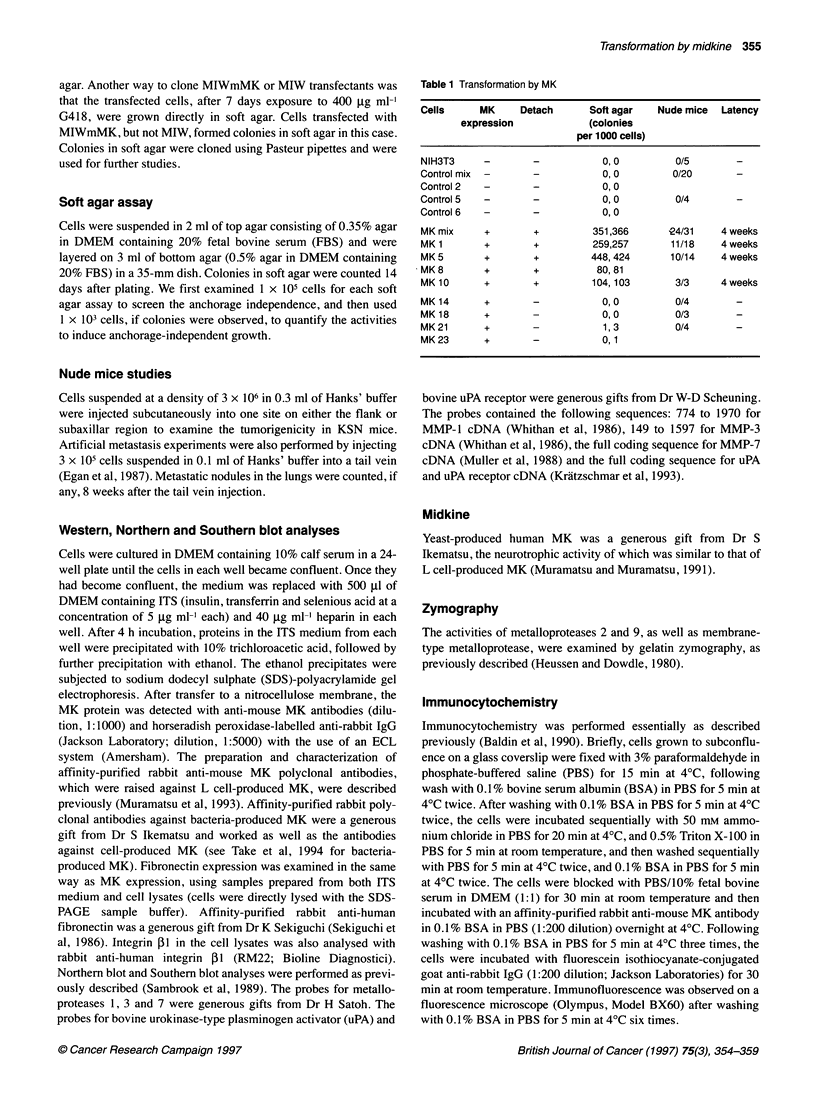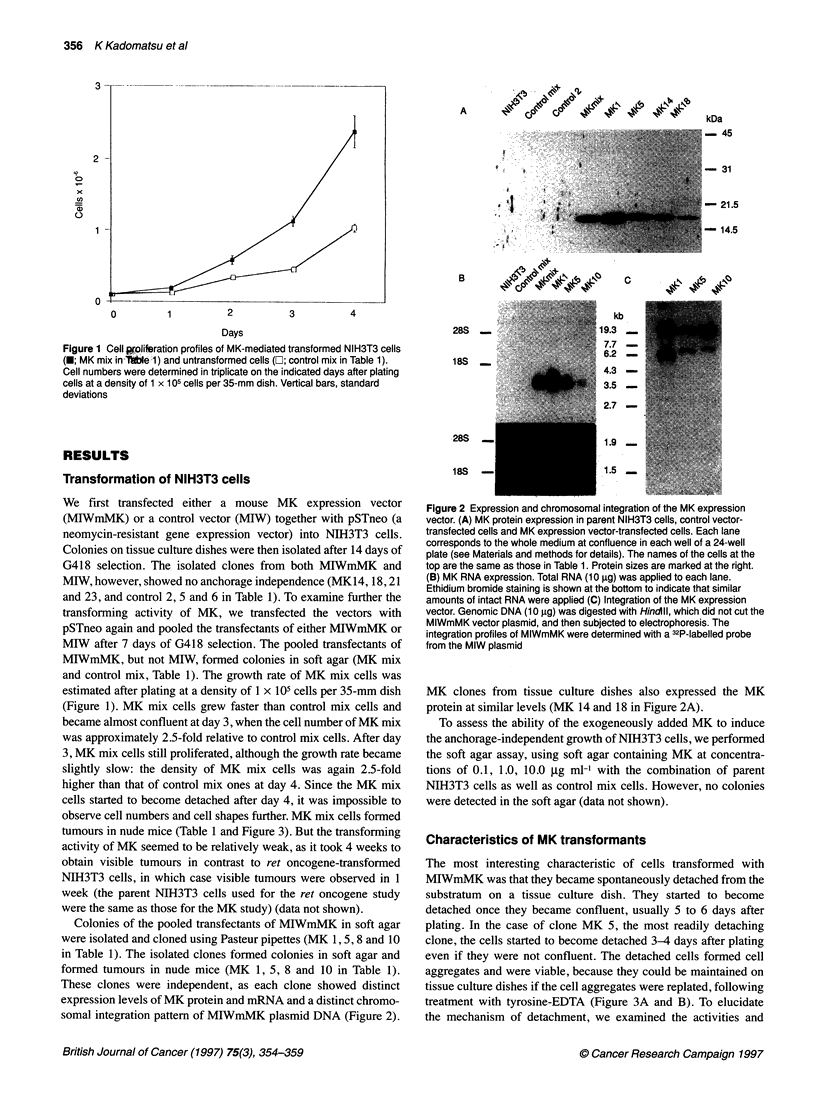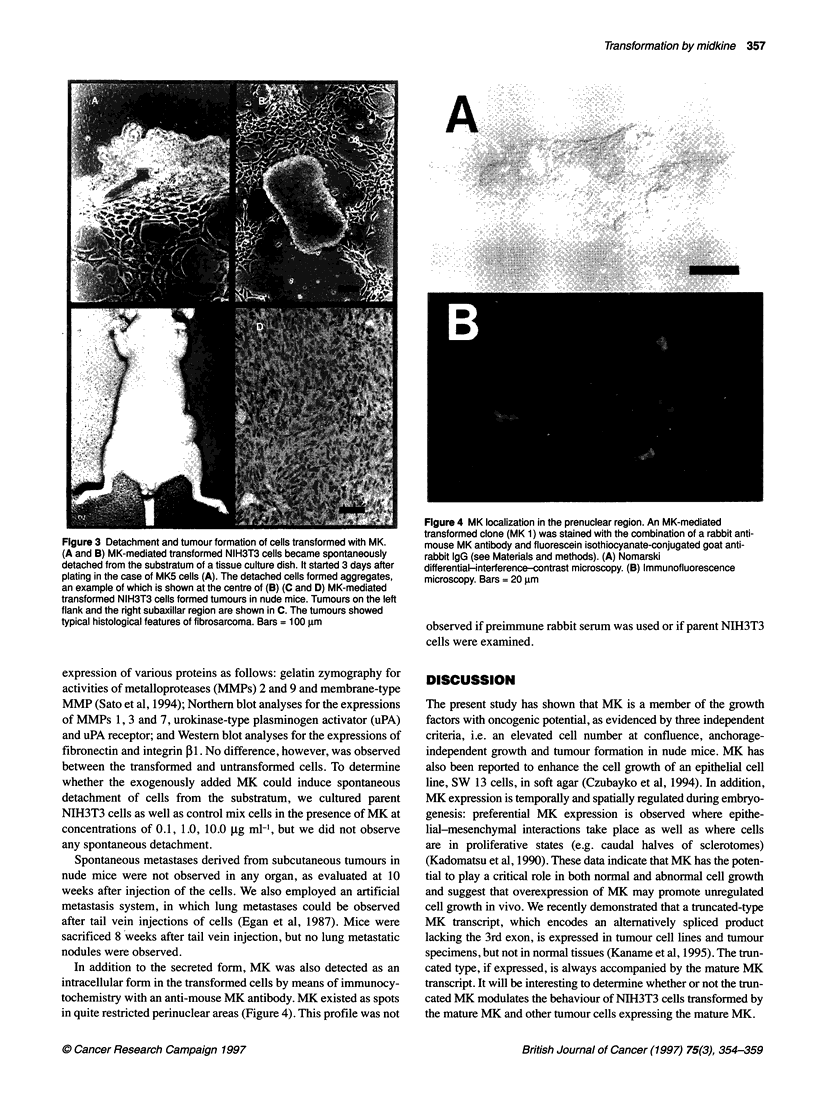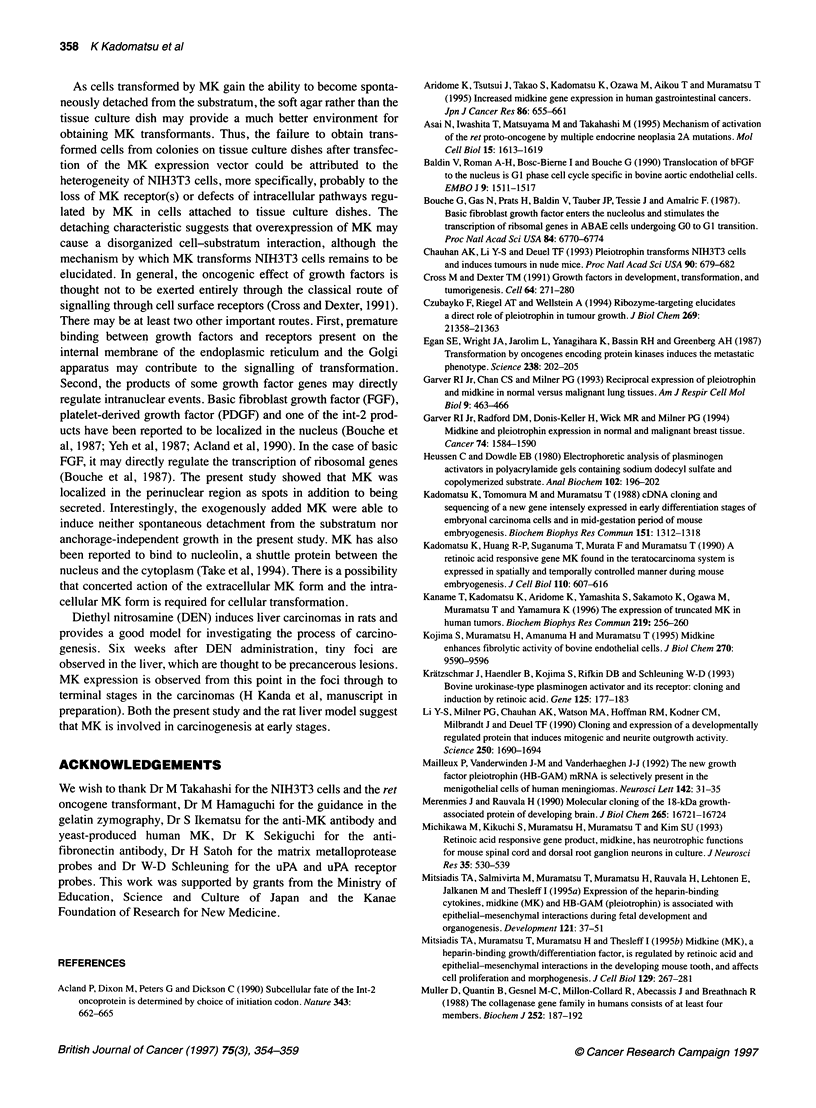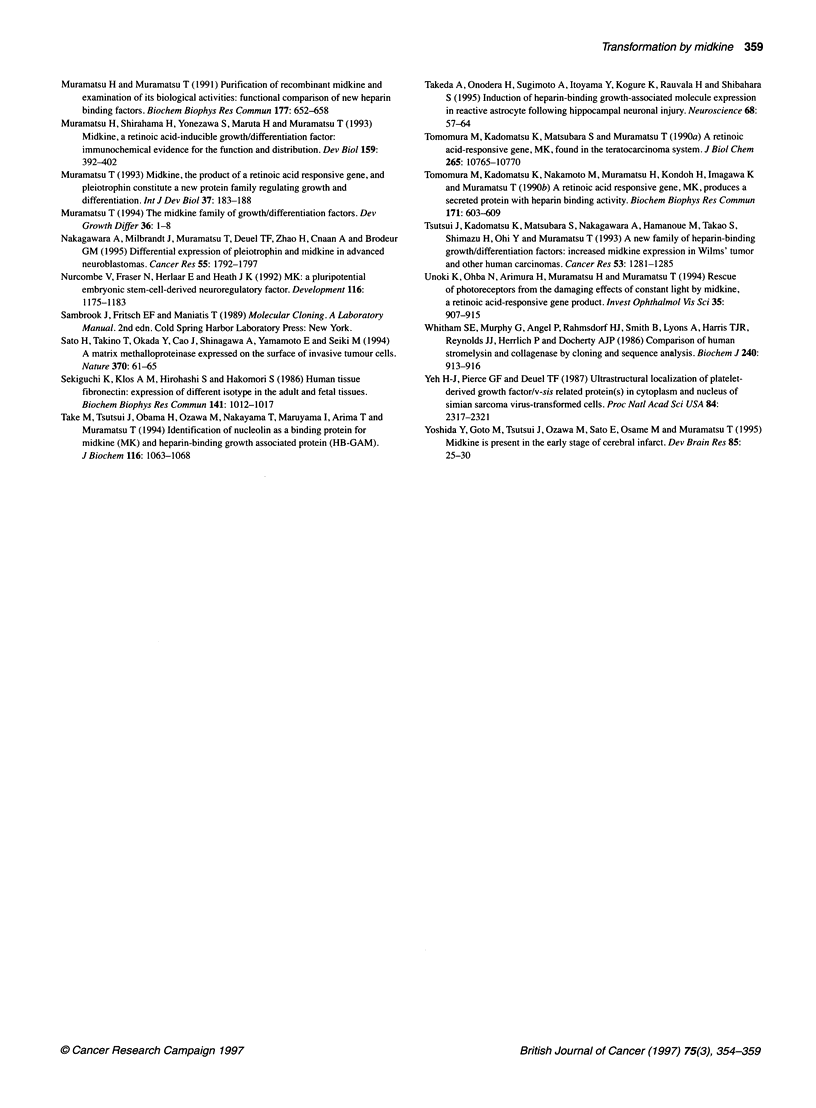# Midkine induces the transformation of NIH3T3 cells.

**DOI:** 10.1038/bjc.1997.58

**Published:** 1997

**Authors:** K. Kadomatsu, M. Hagihara, S. Akhter, Q. W. Fan, H. Muramatsu, T. Muramatsu

**Affiliations:** Department of Biochemistry, Nagoya University School of Medicine, Tsurumai-cho, Showa-ku, Japan.

## Abstract

**Images:**


British Journal of Cancer (1997) 75(3), 354–359
© 1997 Cancer Research Campaign

### Midkine induces the transformation of NIH3T3 cells

**K Kadomatsu, M Hagihara, S Akhter, Q-W Fan, H Muramatsu and T Muramatsu**

Department of Biochemistry, Nagoya University School of Medicine, 65 Tsurumai-cho, Showa-ku, Nagoya 466, Japan

**Summary** Midkine (MK) is a heparin-binding growth factor and is frequently expressed at high levels in many human carcinomas. To investigate further the roles of MK in the regulation of cell growth, we introduced MK expression in NIH3T3 cells. A mixture of transfectants of an MK expression vector, but not a control vector, formed colonies in soft agar, showed an elevated cell number at confluence, and formed tumours in nude mice. An interesting characteristic of the transformed cells was that they became spontaneously detached from the culture dish substratum. In the transformed cells, MK was not only secreted, but also localized, in the perinuclear region as spots. The present data indicate that MK has the potential to transform NIH3T3 cells and suggest that overexpression of the MK gene may promote unregulated cell growth in vivo.

**Keywords:** carcinogenesis; growth factor; midkine; pleiotrophin; transformation

Polypeptide growth factors play regulatory roles in the development and maintenance of normal tissues and also contribute to the processes of transformation and tumorigenesis in vivo (Cross and Dexter, 1991). Midkine (MK), originally isolated as a product of a retinoic acid-responsive gene in an embryonal carcinoma cell differentiation system, is a heparin-binding growth factor (Kadomatsu et al, 1988; Tomomura et al, 1990 *a,b*) implicated in neuronal survival and differentiation (Muramatsu and Muramatsu, 1991; Michikawa et al, 1993; Unoki et al, 1994), carcinogenesis (Tsutsui et al, 1993; Nakagawara et al, 1995), fibrinolysis (Kojima et al, 1995), wound healing (Yoshida et al, 1995) and development (Kadomatsu et al, 1990; Mitsiadis et al, 1995*a*, *b*). MK belongs to a novel growth factor family whose only members so far are MK and pleiotrophin (PTN)/HB-GAM (Muramatsu, 1993, 1994). MK and PTN have approximately 50% homology, with completely conserved cysteine positions, and share several biochemical and biological properties: heparin-binding, neurotrophic activity (Li et al, 1990; Merenmies and Rauvala, 1990) and involvement in wound healing (Takeda et al, 1995) and carcinogenesis (Chauhan et al, 1993).

Expression of MK and PTN has been investigated in many human tumours, including meningiomas (Mailleux et al, 1992), Wilms' tumours (Tsutsui et al, 1993), lung carcinomas (Garver et al, 1993), breast carcinomas (Garver et al, 1994), neuroblastomas (Nakagawara et al, 1995), gastric carcinomas, hepatic carcinomas and colon carcinomas (Aridome et al, 1995). The expression profiles of MK, compared with those of PTN, appear to be more aggressive in general. For example, MK is more frequently and abundantly expressed in Wilms' tumours (Tsutsui et al, 1993). In the lung, MK is not expressed in normal tissues and is abundantly expressed in the carcinomas, but PTN expression is reciprocal (Garver et al, 1993). In neuroblastomas, PTN is expressed in early clinical stages, and there is a reverse relationship between PTN expression and a poor prognosis. MK expression in neuroblastomas is relatively constant through the whole clinical stages and is more abundant than PTN in each stage (Nakagawara et al, 1995). On the other hand, PTN has been reported to transform NIH3T3 cells (Chauhan et al, 1993). Ribozyme-mediated PTN RNA disruption results in a loss of tumorigenecity of WM852 cells (a human melanoma cell line) (Czubayko et al, 1994).

In addition to the preferential expression in human tumours, MK provides growth advantages to some cells: MK enhances the proliferation of NIH3T3 cells by approximately twofold (Muramatsu and Muramatsu, 1991), and of 1009 EC cells by threefold at a differentiation state induced by retinoic acid (Nurcombe et al, 1992); anti-MK antibodies partially inhibit the proliferation of G401 cells (a Wilms' tumour cell line) (Muramatsu et al, 1993). Taken together, all the data described above indicate that MK could be involved in carcinogenesis, but the biological functions of MK, essential for the process of carcinogenesis, have not been determined. In the present study, we focused on investigation of the activities of MK to induce cellular transformation and tumours in nude mice.

### MATERIALS AND METHODS

### Cells and DNA transfection

NIH3T3 cells and *ret* oncogene-transformed NIH3T3 cells were generous gifts from Dr H Takahashi (Asai et al, 1995). Both the NIH3T3 cells and *ret* oncogene-transformed cells were maintained in Dulbecco's modified Eagle medium (DMEM) containing 10% calf serum. For DNA transfection, NIH3T3 cells were plated at a density of $3 \times 10^5$ cells in a 35-mm dish. On the following day, 10 μg of either MIWmMK (a mouse MK expression vector under the control of the Raus sarcoma virus enhancer and the chicken β-actin promoter and enhancer) or MIW (an empty vector without any inserted cDNA) was transfected together with 1 μg of pSTneo (a neomycin-resistant gene expression vector) into NIH3T3 cells by the use of lipofectin (Gibco BRL), following the manufacturer's instruction (for the vectors, see Tomomura et al, 1990*b*). Neomycin-resistant colonies were isolated from the tissue culture dishes after 14 days exposure to 400 μg $ml^{-1}$ G418 (Sigma), followed by examination of anchorage-independent growth in soft

*Received 26 April 1996*
*Revised 28 August 1996*
*Accepted 29 August 1996*

*Correspondence to:* K Kadomatsu agar. Another way to clone MIWmMK or MIW transfectants was that the transfected cells, after 7 days exposure to 400 μg $ml^{-1}$ G418, were grown directly in soft agar. Cells transfected with MIWmMK, but not MIW, formed colonies in soft agar in this case. Colonies in soft agar were cloned using Pasteur pipettes and were used for further studies.

### Soft agar assay

Cells were suspended in 2 ml of top agar consisting of 0.35% agar in DMEM containing 20% fetal bovine serum (FBS) and were layered on 3 ml of bottom agar (0.5% agar in DMEM containing 20% FBS) in a 35-mm dish. Colonies in soft agar were counted 14 days after plating. We first examined $1 \times 10^5$ cells for each soft agar assay to screen the anchorage independence, and then used $1 \times 10^3$ cells, if colonies were observed, to quantify the activities to induce anchorage-independent growth.

### Nude mice studies

Cells suspended at a density of $3 \times 10^6$ in 0.3 ml of Hanks' buffer were injected subcutaneously into one site on either the flank or subaxillar region to examine the tumorigenicity in KSN mice. Artificial metastasis experiments were also performed by injecting $3 \times 10^5$ cells suspended in 0.1 ml of Hanks' buffer into a tail vein (Egan et al, 1987). Metastatic nodules in the lungs were counted, if any, 8 weeks after the tail vein injection.

### Western, Northern and Southern blot analyses

Cells were cultured in DMEM containing 10% calf serum in a 24-well plate until the cells in each well became confluent. Once they had become confluent, the medium was replaced with 500 μl of DMEM containing ITS (insulin, transferrin and selenious acid at a concentration of 5 μg $ml^{-1}$ each) and 40 μg $ml^{-1}$ heparin in each well. After 4 h incubation, proteins in the ITS medium from each well were precipitated with 10% trichloroacetic acid, followed by further precipitation with ethanol. The ethanol precipitates were subjected to sodium dodecyl sulphate (SDS)-polyacrylamide gel electrophoresis. After transfer to a nitrocellulose membrane, the MK protein was detected with anti-mouse MK antibodies (dilution, 1:1000) and horseradish peroxidase-labelled anti-rabbit IgG (Jackson Laboratory; dilution, 1:5000) with the use of an ECL system (Amersham). The preparation and characterization of affinity-purified rabbit anti-mouse MK polyclonal antibodies, which were raised against L cell-produced MK, were described previously (Muramatsu et al, 1993). Affinity-purified rabbit polyclonal antibodies against bacteria-produced MK were a generous gift from Dr S Ikematsu and worked as well as the antibodies against cell-produced MK (see Take et al, 1994 for bacteria-produced MK). Fibronectin expression was examined in the same way as MK expression, using samples prepared from both ITS medium and cell lysates (cells were directly lysed with the SDS-PAGE sample buffer). Affinity-purified rabbit anti-human fibronectin was a generous gift from Dr K Sekiguchi (Sekiguchi et al, 1986). Integrin β1 in the cell lysates was also analysed with rabbit anti-human integrin β1 (RM22; Bioline Diagnostici). Northern blot and Southern blot analyses were performed as previously described (Sambrook et al, 1989). The probes for metalloproteases 1, 3 and 7 were generous gifts from Dr H Satoh. The probes for bovine urokinase-type plasminogen activator (uPA) and bovine uPA receptor were generous gifts from Dr W-D Scheuning. The probes contained the following sequences: 774 to 1970 for MMP-1 cDNA (Whithan et al, 1986), 149 to 1597 for MMP-3 cDNA (Whithan et al, 1986), the full coding sequence for MMP-7 cDNA (Muller et al, 1988) and the full coding sequence for uPA and uPA receptor cDNA (Krätzschmar et al, 1993).

**Table 1** Transformation by MK

| Cells | MK expression | Detach | Soft agar (colonies per 1000 cells) | Nude mice | Latency |
|---|---|---|---|---|---|
| NIH3T3 | – | – | 0, 0 | 0/5 | – |
| Control mix | – | – | 0, 0 | 0/20 | – |
| Control 2 | – | – | 0, 0 | | |
| Control 5 | – | – | 0, 0 | 0/4 | – |
| Control 6 | – | – | 0, 0 | | |
| MK mix | + | + | 351,366 | 24/31 | 4 weeks |
| MK 1 | + | + | 259,257 | 11/18 | 4 weeks |
| MK 5 | + | + | 448, 424 | 10/14 | 4 weeks |
| MK 8 | + | + | 80, 81 | | |
| MK 10 | + | + | 104, 103 | 3/3 | 4 weeks |
| MK 14 | + | – | 0, 0 | 0/4 | – |
| MK 18 | + | – | 0, 0 | 0/3 | – |
| MK 21 | + | – | 1, 3 | 0/4 | – |
| MK 23 | + | – | 0, 1 | | |

### Midkine

Yeast-produced human MK was a generous gift from Dr S Ikematsu, the neurotrophic activity of which was similar to that of L cell-produced MK (Muramatsu and Muramatsu, 1991).

### Zymography

The activities of metalloproteases 2 and 9, as well as membrane-type metalloprotease, were examined by gelatin zymography, as previously described (Heussen and Dowdle, 1980).

### Immunocytochemistry

Immunocytochemistry was performed essentially as described previously (Baldin et al, 1990). Briefly, cells grown to subconfluence on a glass coverslip were fixed with 3% paraformaldehyde in phosphate-buffered saline (PBS) for 15 min at 4°C, following wash with 0.1% bovine serum albumin (BSA) in PBS for 5 min at 4°C twice. After washing with 0.1% BSA in PBS for 5 min at 4°C twice, the cells were incubated sequentially with 50 mM ammonium chloride in PBS for 20 min at 4°C, and 0.5% Triton X-100 in PBS for 5 min at room temperature, and then washed sequentially with PBS for 5 min at 4°C twice, and 0.1% BSA in PBS for 5 min at 4°C twice. The cells were blocked with PBS/10% fetal bovine serum in DMEM (1:1) for 30 min at room temperature and then incubated with an affinity-purified rabbit anti-mouse MK antibody in 0.1% BSA in PBS (1:200 dilution) overnight at 4°C. Following washing with 0.1% BSA in PBS for 5 min at 4°C three times, the cells were incubated with fluorescein isothiocyanate-conjugated goat anti-rabbit IgG (1:200 dilution; Jackson Laboratories) for 30 min at room temperature. Immunofluorescence was observed on a fluorescence microscope (Olympus, Model BX60) after washing with 0.1% BSA in PBS for 5 min at 4°C six times.

**Figure 1** Cell proliferation profiles of MK-mediated transformed NIH3T3 cells (■; MK mix in Table 1) and untransformed cells (□; control mix in Table 1). Cell numbers were determined in triplicate on the indicated days after plating cells at a density of 1 × $10^5$ cells per 35-mm dish. Vertical bars, standard deviations

**Figure 2** Expression and chromosomal integration of the MK expression vector. (**A**) MK protein expression in parent NIH3T3 cells, control vector-transfected cells and MK expression vector-transfected cells. Each lane corresponds to the whole medium at confluence in each well of a 24-well plate (see Materials and methods for details). The names of the cells at the top are the same as those in Table 1. Protein sizes are marked at the right. (**B**) MK RNA expression. Total RNA (10 μg) was applied to each lane. Ethidium bromide staining is shown at the bottom to indicate that similar amounts of intact RNA were applied (**C**) Integration of the MK expression vector. Genomic DNA (10 μg) was digested with *Hin*dIII, which did not cut the MIWmMK vector plasmid, and then subjected to electrophoresis. The integration profiles of MIWmMK were determined with a $^{32}$P-labelled probe from the MIW plasmid

### RESULTS

### Transformation of NIH3T3 cells

We first transfected either a mouse MK expression vector (MIWmMK) or a control vector (MIW) together with pSTneo (a neomycin-resistant gene expression vector) into NIH3T3 cells. Colonies on tissue culture dishes were then isolated after 14 days of G418 selection. The isolated clones from both MIWmMK and MIW, however, showed no anchorage independence (MK14, 18, 21 and 23, and control 2, 5 and 6 in Table 1). To examine further the transforming activity of MK, we transfected the vectors with pSTneo again and pooled the transfectants of either MIWmMK or MIW after 7 days of G418 selection. The pooled transfectants of MIWmMK, but not MIW, formed colonies in soft agar (MK mix and control mix, Table 1). The growth rate of MK mix cells was estimated after plating at a density of 1 × $10^5$ cells per 35-mm dish (Figure 1). MK mix cells grew faster than control mix cells and became almost confluent at day 3, when the cell number of MK mix was approximately 2.5-fold relative to control mix cells. After day 3, MK mix cells still proliferated, although the growth rate became slightly slow: the density of MK mix cells was again 2.5-fold higher than that of control mix ones at day 4. Since the MK mix cells started to become detached after day 4, it was impossible to observe cell numbers and cell shapes further. MK mix cells formed tumours in nude mice (Table 1 and Figure 3). But the transforming activity of MK seemed to be relatively weak, as it took 4 weeks to obtain visible tumours in contrast to *ret* oncogene-transformed NIH3T3 cells, in which case visible tumours were observed in 1 week (the parent NIH3T3 cells used for the *ret* oncogene study were the same as those for the MK study) (data not shown).

Colonies of the pooled transfectants of MIWmMK in soft agar were isolated and cloned using Pasteur pipettes (MK 1, 5, 8 and 10 in Table 1). The isolated clones formed colonies in soft agar and formed tumours in nude mice (MK 1, 5, 8 and 10 in Table 1). These clones were independent, as each clone showed distinct expression levels of MK protein and mRNA and a distinct chromosomal integration pattern of MIWmMK plasmid DNA (Figure 2).

MK clones from tissue culture dishes also expressed the MK protein at similar levels (MK 14 and 18 in Figure 2A).

To assess the ability of the exogeneously added MK to induce the anchorage-independent growth of NIH3T3 cells, we performed the soft agar assay, using soft agar containing MK at concentrations of 0.1, 1.0, 10.0 μg $ml^{-1}$ with the combination of parent NIH3T3 cells as well as control mix cells. However, no colonies were detected in the soft agar (data not shown).

### Characteristics of MK transformants

The most interesting characteristic of cells transformed with MIWmMK was that they became spontaneously detached from the substratum on a tissue culture dish. They started to become detached once they became confluent, usually 5 to 6 days after plating. In the case of clone MK 5, the most readily detaching clone, the cells started to become detached 3–4 days after plating even if they were not confluent. The detached cells formed cell aggregates and were viable, because they could be maintained on tissue culture dishes if the cell aggregates were replated, following treatment with tyrosine-EDTA (Figure 3A and B). To elucidate the mechanism of detachment, we examined the activities and

© Cancer Research Campaign 1997

**Figure 3** Detachment and tumour formation of cells transformed with MK. (**A** and **B**) MK-mediated transformed NIH3T3 cells became spontaneously detached from the substratum of a tissue culture dish. It started 3 days after plating in the case of MK5 cells (**A**). The detached cells formed aggregates, an example of which is shown at the centre of (**B**) (**C** and **D**) MK-mediated transformed NIH3T3 cells formed tumours in nude mice. Tumours on the left flank and the right subaxillar region are shown in **C**. The tumours showed typical histological features of fibrosarcoma. Bars = 100 μm

**Figure 4** MK localization in the prenuclear region. An MK-mediated transformed clone (MK 1) was stained with the combination of a rabbit anti-mouse MK antibody and fluorescein isothiocyanate-conjugated goat anti-rabbit IgG (see Materials and methods). (**A**) Nomarski differential–interference–contrast microscopy. (**B**) Immunofluorescence microscopy. Bars = 20 μm expression of various proteins as follows: gelatin zymography for activities of metalloproteases (MMPs) 2 and 9 and membrane-type MMP (Sato et al, 1994); Northern blot analyses for the expressions of MMPs 1, 3 and 7, urokinase-type plasminogen activator (uPA) and uPA receptor; and Western blot analyses for the expressions of fibronectin and integrin β1. No difference, however, was observed between the transformed and untransformed cells. To determine whether the exogenously added MK could induce spontaneous detachment of cells from the substratum, we cultured parent NIH3T3 cells as well as control mix cells in the presence of MK at concentrations of 0.1, 1.0, 10.0 μg $ml^{-1}$, but we did not observe any spontaneous detachment.

Spontaneous metastases derived from subcutaneous tumours in nude mice were not observed in any organ, as evaluated at 10 weeks after injection of the cells. We also employed an artificial metastasis system, in which lung metastases could be observed after tail vein injections of cells (Egan et al, 1987). Mice were sacrificed 8 weeks after tail vein injection, but no lung metastatic nodules were observed.

In addition to the secreted form, MK was also detected as an intracellular form in the transformed cells by means of immunocytochemistry with an anti-mouse MK antibody. MK existed as spots in quite restricted perinuclear areas (Figure 4). This profile was not observed if preimmune rabbit serum was used or if parent NIH3T3 cells were examined.

### DISCUSSION

The present study has shown that MK is a member of the growth factors with oncogenic potential, as evidenced by three independent criteria, i.e. an elevated cell number at confluence, anchorage-independent growth and tumour formation in nude mice. MK has also been reported to enhance the cell growth of an epithelial cell line, SW 13 cells, in soft agar (Czubayko et al, 1994). In addition, MK expression is temporally and spatially regulated during embryogenesis: preferential MK expression is observed where epithelial–mesenchymal interactions take place as well as where cells are in proliferative states (e.g. caudal halves of sclerotomes) (Kadomatsu et al, 1990). These data indicate that MK has the potential to play a critical role in both normal and abnormal cell growth and suggest that overexpression of MK may promote unregulated cell growth in vivo. We recently demonstrated that a truncated-type MK transcript, which encodes an alternatively spliced product lacking the 3rd exon, is expressed in tumour cell lines and tumour specimens, but not in normal tissues (Kaname et al, 1995). The truncated type, if expressed, is always accompanied by the mature MK transcript. It will be interesting to determine whether or not the truncated MK modulates the behaviour of NIH3T3 cells transformed by the mature MK and other tumour cells expressing the mature MK.

As cells transformed by MK gain the ability to become spontaneously detached from the substratum, the soft agar rather than the tissue culture dish may provide a much better environment for obtaining MK transformants. Thus, the failure to obtain transformed cells from colonies on tissue culture dishes after transfection of the MK expression vector could be attributed to the heterogeneity of NIH3T3 cells, more specifically, probably to the loss of MK receptor(s) or defects of intracellular pathways regulated by MK in cells attached to tissue culture dishes. The detaching characteristic suggests that overexpression of MK may cause a disorganized cell–substratum interaction, although the mechanism by which MK transforms NIH3T3 cells remains to be elucidated. In general, the oncogenic effect of growth factors is thought not to be exerted entirely through the classical route of signalling through cell surface receptors (Cross and Dexter, 1991). There may be at least two other important routes. First, premature binding between growth factors and receptors present on the internal membrane of the endoplasmic reticulum and the Golgi apparatus may contribute to the signalling of transformation. Second, the products of some growth factor genes may directly regulate intranuclear events. Basic fibroblast growth factor (FGF), platelet-derived growth factor (PDGF) and one of the int-2 products have been reported to be localized in the nucleus (Bouche et al, 1987; Yeh et al, 1987; Acland et al, 1990). In the case of basic FGF, it may directly regulate the transcription of ribosomal genes (Bouche et al, 1987). The present study showed that MK was localized in the perinuclear region as spots in addition to being secreted. Interestingly, the exogenously added MK were able to induce neither spontaneous detachment from the substratum nor anchorage-independent growth in the present study. MK has also been reported to bind to nucleolin, a shuttle protein between the nucleus and the cytoplasm (Take et al, 1994). There is a possibility that concerted action of the extracellular MK form and the intracellular MK form is required for cellular transformation.

Diethyl nitrosamine (DEN) induces liver carcinomas in rats and provides a good model for investigating the process of carcinogenesis. Six weeks after DEN administration, tiny foci are observed in the liver, which are thought to be precancerous lesions. MK expression is observed from this point in the foci through to terminal stages in the carcinomas (H Kanda et al, manuscript in preparation). Both the present study and the rat liver model suggest that MK is involved in carcinogenesis at early stages.

### ACKNOWLEDGEMENTS

We wish to thank Dr M Takahashi for the NIH3T3 cells and the *ret* oncogene transformant, Dr M Hamaguchi for the guidance in the gelatin zymography, Dr S Ikematsu for the anti-MK antibody and yeast-produced human MK, Dr K Sekiguchi for the anti-fibronectin antibody, Dr H Satoh for the matrix metalloprotease probes and Dr W-D Schleuning for the uPA and uPA receptor probes. This work was supported by grants from the Ministry of Education, Science and Culture of Japan and the Kanae Foundation of Research for New Medicine.

### REFERENCES

Acland P, Dixon M, Peters G and Dickson C (1990) Subcellular fate of the Int-2 oncoprotein is determined by choice of initiation codon. *Nature* **343**: 662–665

Aridome K, Tsutsui J, Takao S, Kadomatsu K, Ozawa M, Aikou T and Muramatsu T (1995) Increased midkine gene expression in human gastrointestinal cancers. *Jpn J Cancer Res* **86**: 655–661

Asai N, Iwashita T, Matsuyama M and Takahashi M (1995) Mechanism of activation of the *ret* proto-oncogene by multiple endocrine neoplasia 2A mutations. *Mol Cell Biol* **15**: 1613–1619

Baldin V, Roman A-H, Bosc-Bierne I and Bouche G (1990) Translocation of bFGF to the nucleus is G1 phase cell cycle specific in bovine aortic endothelial cells. *EMBO J* **9**: 1511–1517

Bouche G, Gas N, Prats H, Baldin V, Tauber JP, Tessie J and Amalric F. (1987). Basic fibroblast growth factor enters the nucleolus and stimulates the transcription of ribsomal genes in ABAE cells undergoing G0 to G1 transition. *Proc Natl Acad Sci USA* **84**: 6770–6774

Chauhan AK, Li Y-S and Deuel TF (1993) Pleiotrophin transforms NIH3T3 cells and induces tumours in nude mice. *Proc Natl Acad Sci USA* **90**: 679–682

Cross M and Dexter TM (1991) Growth factors in development, transformation, and tumorigenesis. *Cell* **64**: 271–280

Czubayko F, Riegel AT and Wellstein A (1994) Ribozyme-targeting elucidates a direct role of pleiotrophin in tumour growth. *J Biol Chem* **269**: 21358–21363

Egan SE, Wright JA, Jarolim L, Yanagihara K, Bassin RH and Greenberg AH (1987) Transformation by oncogenes encoding protein kinases induces the metastatic phenotype. *Science* **238**: 202–205

Garver RI Jr, Chan CS and Milner PG (1993) Reciprocal expression of pleiotrophin and midkine in normal versus malignant lung tissues. *Am J Respir Cell Mol Biol* **9**: 463–466

Garver RI Jr, Radford DM, Donis-Keller H, Wick MR and Milner PG (1994) Midkine and pleiotrophin expression in normal and malignant breast tissue. *Cancer* **74**: 1584–1590

Heussen C and Dowdle EB (1980) Electrophoretic analysis of plasminogen activators in polyacrylamide gels containing sodium dodecyl sulfate and copolymerized substrate. *Anal Biochem* **102**: 196–202

Kadomatsu K, Tomomura M and Muramatsu T (1988) cDNA cloning and sequencing of a new gene intensely expressed in early differentiation stages of embryonal carcinoma cells and in mid-gestation period of mouse embryogenesis. *Biochem Biophys Res Commun* **151**: 1312–1318

Kadomatsu K, Huang R-P, Suganuma T, Murata F and Muramatsu T (1990) A retinoic acid responsive gene MK found in the teratocarcinoma system is expressed in spatially and temporally controlled manner during mouse embryogenesis. *J Cell Biol* **110**: 607–616

Kaname T, Kadomatsu K, Aridome K, Yamashita S, Sakamoto K, Ogawa M, Muramatsu T and Yamamura K (1996) The expression of truncated MK in human tumors. *Biochem Biophys Res Commun* **219**: 256–260

Kojima S, Muramatsu H, Amanuma H and Muramatsu T (1995) Midkine enhances fibrolytic activity of bovine endothelial cells. *J Biol Chem* **270**: 9590–9596

Krätzschmar J, Haendler B, Kojima S, Rifkin DB and Schleuning W-D (1993) Bovine urokinase-type plasminogen activator and its receptor: cloning and induction by retinoic acid. *Gene* **125**: 177–183

Li Y-S, Milner PG, Chauhan AK, Watson MA, Hoffman RM, Kodner CM, Milbrandt J and Deuel TF (1990) Cloning and expression of a developmentally regulated protein that induces mitogenic and neurite outgrowth activity. *Science* **250**: 1690–1694

Mailleux P, Vanderwinden J-M and Vanderhaeghen J-J (1992) The new growth factor pleiotrophin (HB-GAM) mRNA is selectively present in the menigothelial cells of human meningiomas. *Neurosci Lett* **142**: 31–35

Merenmies J and Rauvala H (1990) Molecular cloning of the 18-kDa growth-associated protein of developing brain. *J Biol Chem* **265**: 16721–16724

Michikawa M, Kikuchi S, Muramatsu H, Muramatsu T and Kim SU (1993) Retinoic acid responsive gene product, midkine, has neurotrophic functions for mouse spinal cord and dorsal root ganglion neurons in culture. *J Neurosci Res* **35**: 530–539

Mitsiadis TA, Salmivirta M, Muramatsu T, Muramatsu H, Rauvala H, Lehtonen E, Jalkanen M and Thesleff I (1995*a*) Expression of the heparin-binding cytokines, midkine (MK) and HB-GAM (pleiotrophin) is associated with epithelial–mesenchymal interactions during fetal development and organogenesis. *Development* **121**: 37–51

Mitsiadis TA, Muramatsu T, Muramatsu H and Thesleff I (1995*b*) Midkine (MK), a heparin-binding growth/differentiation factor, is regulated by retinoic acid and epithelial–mesenchymal interactions in the developing mouse tooth, and affects cell proliferation and morphogenesis. *J Cell Biol* **129**: 267–281

Muller D, Quantin B, Gesnel M-C, Millon-Collard R, Abecassis J and Breathnach R (1988) The collagenase gene family in humans consists of at least four members. *Biochem J* **252**: 187–192

© Cancer Research Campaign 1997

Muramatsu H and Muramatsu T (1991) Purification of recombinant midkine and examination of its biological activities: functional comparison of new heparin binding factors. *Biochem Biophys Res Commun* **177**: 652–658

Muramatsu H, Shirahama H, Yonezawa S, Maruta H and Muramatsu T (1993) Midkine, a retinoic acid-inducible growth/differentiation factor: immunochemical evidence for the function and distribution. *Dev Biol* **159**: 392–402

Muramatsu T (1993) Midkine, the product of a retinoic acid responsive gene, and pleiotrophin constitute a new protein family regulating growth and differentiation. *Int J Dev Biol* **37**: 183–188

Muramatsu T (1994) The midkine family of growth/differentiation factors. *Dev Growth Differ* **36**: 1–8

Nakagawara A, Milbrandt J, Muramatsu T, Deuel TF, Zhao H, Cnaan A and Brodeur GM (1995) Differential expression of pleiotrophin and midkine in advanced neuroblastomas. *Cancer Res* **55**: 1792–1797

Nurcombe V, Fraser N, Herlaar E and Heath J K (1992) MK: a pluripotential embryonic stem-cell-derived neuroregulatory factor. *Development* **116**: 1175–1183

Sambrook J, Fritsch EF and Maniatis T (1989) *Molecular Cloning. A Laboratory Manual*. 2nd edn. Cold Spring Harbor Laboratory Press: New York.

Sato H, Takino T, Okada Y, Cao J, Shinagawa A, Yamamoto E and Seiki M (1994) A matrix methalloproteinase expressed on the surface of invasive tumour cells. *Nature* **370**: 61–65

Sekiguchi K, Klos A M, Hirohashi S and Hakomori S (1986) Human tissue fibronectin: expression of different isotype in the adult and fetal tissues. *Biochem Biophys Res Commun* **141**: 1012–1017

Take M, Tsutsui J, Obama H, Ozawa M, Nakayama T, Maruyama I, Arima T and Muramatsu T (1994) Identification of nucleolin as a binding protein for midkine (MK) and heparin-binding growth associated protein (HB-GAM). *J Biochem* **116**: 1063–1068

Takeda A, Onodera H, Sugimoto A, Itoyama Y, Kogure K, Rauvala H and Shibahara S (1995) Induction of heparin-binding growth-associated molecule expression in reactive astrocyte following hippocampal neuronal injury. *Neuroscience* **68**: 57–64

Tomomura M, Kadomatsu K, Matsubara S and Muramatsu T (1990*a*) A retinoic acid-responsive gene, MK, found in the teratocarcinoma system. *J Biol Chem* **265**: 10765–10770

Tomomura M, Kadomatsu K, Nakamoto M, Muramatsu H, Kondoh H, Imagawa K and Muramatsu T (1990*b*) A retinoic acid responsive gene, MK, produces a secreted protein with heparin binding activity. *Biochem Biophys Res Commun* **171**: 603–609

Tsutsui J, Kadomatsu K, Matsubara S, Nakagawara A, Hamanoue M, Takao S, Shimazu H, Ohi Y and Muramatsu T (1993) A new family of heparin-binding growth/differentiation factors: increased midkine expression in Wilms' tumor and other human carcinomas. *Cancer Res* **53**: 1281–1285

Unoki K, Ohba N, Arimura H, Muramatsu H and Muramatsu T (1994) Rescue of photoreceptors from the damaging effects of constant light by midkine, a retinoic acid-responsive gene product. *Invest Ophthalmol Vis Sci* **35**: 907–915

Whitham SE, Murphy G, Angel P, Rahmsdorf HJ, Smith B, Lyons A, Harris TJR, Reynolds JJ, Herrlich P and Docherty AJP (1986) Comparison of human stromelysin and collagenase by cloning and sequence analysis. *Biochem J* **240**: 913–916

Yeh H-J, Pierce GF and Deuel TF (1987) Ultrastructural localization of platelet-derived growth factor/v-*sis* related protein(s) in cytoplasm and nucleus of simian sarcoma virus-transformed cells. *Proc Natl Acad Sci USA* **84**: 2317–2321

Yoshida Y, Goto M, Tsutsui J, Ozawa M, Sato E, Osame M and Muramatsu T (1995) Midkine is present in the early stage of cerebral infarct. *Dev Brain Res* **85**: 25–30

© Cancer Research Campaign 1997